# Label-Free Differentiation of Cancer and Non-Cancer Cells Based on Machine-Learning-Algorithm-Assisted Fast Raman Imaging

**DOI:** 10.3390/bios12040250

**Published:** 2022-04-15

**Authors:** Qing He, Wen Yang, Weiquan Luo, Stefan Wilhelm, Binbin Weng

**Affiliations:** 1 School of Electrical and Computer Engineering, University of Oklahoma, Norman, OK 73072, USA; 2 Stephenson School of Biomedical Engineering, University of Oklahoma, Norman, OK 73072, USA; weny@ou.edu (W.Y.); stefan.wilhelm@ou.edu (S.W.); 3 Agricultural and Biosystems Engineering, Iowa State University, Ames, IA 50010, USA; weiquanl@iastate.edu

**Keywords:** Raman spectroscopy, PCA, machine learning, non-invasive imaging, fast Raman imaging, cancer cells

## Abstract

This paper proposes a rapid, label-free, and non-invasive approach for identifying murine cancer cells (B16F10 melanoma cancer cells) from non-cancer cells (C2C12 muscle cells) using machine-learning-assisted Raman spectroscopic imaging. Through quick Raman spectroscopic imaging, a hyperspectral data processing approach based on machine learning methods proved capable of presenting the cell structure and distinguishing cancer cells from non-cancer muscle cells without compromising full-spectrum information. This study discovered that biomolecular information–nucleic acids, proteins, and lipids—from cells could be retrieved efficiently from low-quality hyperspectral Raman datasets and then employed for cell line differentiation.

## 1. Introduction

Melanoma is the most prevalent type of skin cancer, accounting for the vast majority of skin-cancer-related mortality in people under the age of 30. While typical malignant melanoma in basal and squamous cells seldom spreads, the less common malignant melanoma is very aggressive and spreads rapidly. The majority of melanoma patients who die have numerous organ metastases. The median survival time for patients diagnosed with distant metastatic melanoma is only 6–10 months, and the 5-year survival rate is only about 6% [1]. Moreover, the autopsy observations imply that only a fraction of the patients with muscle metastasis survive long enough for clinical detection. Thereby, an efficient early screening method is crucial to improve the patients’ survival rate. However, by far, the mass in skeletal muscle caused by metastatic carcinoma could often be misdiagnosed as soft tissue sarcoma [2]. Part of the difficulties in differentiating among carcinomas, sarcomas, melanomas, or other muscle disorders lie in the lack of specificity with developed imaging studies such as computed tomography (CT) scanning and magnetic resonance imaging (MRI) [3].

Recently, due to the time- and labor-intensive procedure of the traditional standard histopathological diagnostic techniques, there has been a rise in interest in clinical spectroscopy for cancer diagnosis. Furthermore, traditional methods may induce artifacts that restrict the interpretation of the data. These complications may cause patients’ prompt diagnosis and surgical therapy to be delayed. Consequently, many optical sensors have been developed to improve the accuracy and efficiency in cancer diagnosis [4], including resonance-based optical sensors [5], fiber-based sensors [6,7], colorimetric biosensors [8], fluorescence-based biosensors [9], and surface-enhanced Raman-spectroscopy (SERS)-based biosensors [10,11]. These sensors are highly sensitive; however, the optimal specificity of these biosensors may rely on the labeling molecules such as aptamers, microRNAs, antibodies, etc., which requires additional sample preparations and could potentially be invasive.

Raman spectroscopy is a well-known alternative that can provide label-free, non-invasive technique for diagnosis detection in multiple fields [11], including virus detection [12], bacteria detection [13,14,15,16,17,18], fungal detection [19], cancer detection [20], etc. Additionally, Raman imaging has drawn great attention in the medical and biology fields due to its potential in proving detailed chemical imaging information of multiple biomolecules simultaneously. Raman imaging has been applied in high-resolution imaging of plant cell wall and human tissues [21] and unprocessed living cells [22]. Thereby, Raman spectroscopic imagining is a promising tool for in vivo and in situ melanoma cancer detection, imaging, and further analysis. However, it is known that Raman signals of most biomolecules are fundamentally weak; only 1 out of 100 million photons would undergo Raman scattering [23]. High-resolution biomolecules’ information signals usually require a long acquisition time and/or high excitation laser power. The data acquisition time for single-spectrum acquisition ranges from 5 to 60 s [13,14,15,16,17,18] depending on the material of interest, sample preparation methods, excitation laser power, Raman system used, etc. This unfortunately limits the application of in situ sample imaging, which consists of thousands of spectra for Raman imaging. Moreover, the extended data acquisition time makes it challenging to image the dynamics of living organisms. Therefore, a robust fast Raman imaging technique for biological samples’ imaging is highly desired.

By far, different techniques have been proposed to improve the imaging speed, including wide-field imaging [24], stimulated Raman scattering (SRS) [25], and coherent anti-Stokes Raman scattering (CARS) microscopy [26]. However, these methods suffer from the limited spectral window, which sacrifices the full-spectrum information. The narrow spectral window hinders the following multivariate analyses for accurate biomolecule content estimation and in-depth information extraction. Nevertheless, the biomolecules in the cellular systems, tissues, and extracellular fluids generally share the same Raman bands [27]. This results in the highly complex spectral information comprised of covariate components corresponding to the abundant and heterogeneous biomolecules in organisms, which leads to inaccurate estimation of biomolecule content estimation based on single-Raman-band intensity. Thus, data mining techniques have been combined with full-spectrum Raman imaging for pattern recognition and information extraction [27,28,29] to decode the complex and information-rich hyperspectral Raman datasets. However, these methods rely on high-quality full-spectrum Raman imaging datasets, which may be challenging to obtain under in vivo and in situ conditions with Raman systems of limited functions and/or limited Raman acquisition time. As a result, to lay a solid basis for an approach dealing with full-spectrum Raman imaging datasets obtained with restricted resources in vivo or in situ, we developed a machine-learning-assisted Raman imaging technique targeting the noisy full-Raman-spectrum dataset obtained with time constrained or low excitation laser energy collected with conventional confocal Raman spectroscopy or portable Raman systems.

In this work, we successfully illustrated the cell structure and differentiated melanoma cancer and muscle cells based on low-quality Raman datasets collected from conventional confocal Raman spectroscopy. Two cell lines that are commonly used as melanoma and muscle models were selected in this study. The murine B16F10 melanoma cell line is the B16 model that is commonly used as the metastatic melanoma model for preclinical studies. The C2C12 cell, which is an immortalized murine myoblast cell line, has been developed for in vitro studies of myoblasts. We demonstrate that this new technique shortens the full-spectrum acquisition time and extracts multivariate information from the dataset with a low signal-to-noise ratio (SNR). The biomolecular information of the cellular systems can then be quickly extracted from the low-quality hyperspectral Raman imaging dataset through data pre-processing and multivariate analysis. The machine learning algorithms are further introduced to build the models and differentiate cancer and non-cancer cells.

## 2. Materials’ Preparation and Analytical Methods

### 2.1. Cell Culturing Process

Two cell line models were selected in this study. The murine B16F10 cell line and C2C12 cell line were provided by Dr. Darrell Irvine. The murine B16F10 melanoma cell line is commonly used as a metastatic melanoma model for preclinical studies. The C2C12 cell line is an immortalized murine myoblast cell line that has been developed for in vitro studies of myoblasts. C2C12 muscle cells, B16F10 cells, and their mixture (C2C12 and B16F10 at a ratio of 2:1) were cultured in the presence of recommended complete media, Dulbecco Modified Eagle’s Medium (DMEM, GIBOCO Lot #2358592), containing 10% fetal bovine serum (cat. 16000-044, Life Technologies, Carlsbad, CA, USA), and 1% pen-strep (cat. 15140-122, Life Technologies, Carlsbad, CA, USA) at 37 °C with 5% CO_2_. Cells were seeded on disinfected glasses within 6-well plates at a density of 5 × 10^4^ cells/well overnight. After overnight incubation, media were aspirated, and cells were then washed by 1x PBS before Raman characterization. The optical images of cell cultures are shown in Figure S1.

### 2.2. Confocal Raman Spectroscopy Measurement

Hyperspectral Raman images were acquired by the Renishaw InVia Reflex Raman mapping microscope system (Renishaw, London, U.K.) coupled with a 532 nm wavelength, 100 mW diode laser. The exaction laser beam was focused into a 50× Leica objective lens for spectral measurement. The cell-seeded glass slides were immersed in PBS buffer for Raman imaging. A 2400 grooves per centimeter grating centered at 1100 cm${}^{-1}$ was used to collect the Raman spectra between 466 cm${}^{-1}$ and 1667 cm${}^{-1}$. All Raman imaging datasets were collected at 100% laser power for 1 s per pixel with a 2 μm step size, each pixel containing a Raman spectrum of the examined pixel that covered the area of 4 μm. Before the experiment, images of the scanned area were captured by optical microscopy, and the wavelength was calibrated by the 520.5 cm${}^{-1}$ silicon signature band as the standard. The collected hyperspectral Raman dataset consisted of 4728 spectra collected from B16F10 cell samples and 2394 spectra from C2C12 cell samples, each spectrum consisting of intensities at 1022 Raman shifts. The spectra collected from PBS buffer background (4592 spectra), B16F10 (2539 spectra), and C2C12 cells (880 cells) were manually labeled based on the optical images. The noisy hyperspectral Raman imaging datasets caused by the short exposure time were collected to mimic the Raman datasets collected under in situ or in vivo circumstances. To verify the data processing result, high-quality spectra were collected from B16F10, cell2, and PBS buffer background with 50% excitation laser power at 50 mW to prevent overheating of samples after the long acquisition time [30,31], and the acquisition time was up to 20 s.

### 2.3. Data Processing Workflow

**Figure 1:**
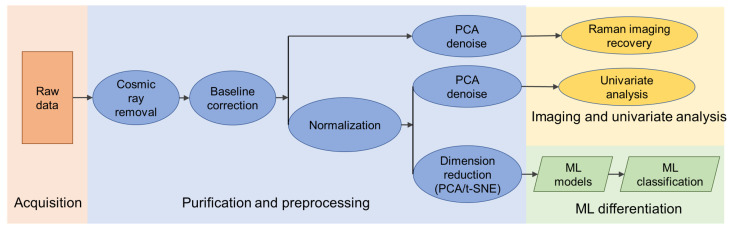
Data processing workflow.

We collected the Raman images from murine skin cancer cells B16F10 and murine healthy muscle cells C2C12 with a short acquisition time. A data analysis workflow designed for the low-quality Raman spectra was applied to the collected dataset. The data analysis flowchart is shown in Figure 1. The dataset was first purified and pre-processed to remove the spectra contaminants including cosmic rays, background fluorescence, and random noise. The following Raman image reconstruction, univariate analysis, and machine learning classifications were further conducted based on the pre-processed dataset.

The contaminants from cosmic rays and background fluorescence that commonly exist in biological samples were removed. Both cosmic rays and background fluorescence would severely distort the Raman spectra and hinder the univariate analysis to quantify the biomolecules of interest through their signature Raman bands or multivariate analysis for deeper data mining. Firstly, The cosmic ray removal was conducted with the WiRE4.2 software package. All the subsequent data analysis were conducted in the R 4.1.2 environment. The polynomial baseline correction was then conducted for each spectrum to remove the Raman spectra contaminant from the background fluorescence. The purified spectra were further analyzed with principal component analysis (PCA) for spectral denoising and high-quality Raman image recovery.

PCA is an unsupervised orthogonal transformation that projects the original dataset into individual linearly independent dimensions. Each unit vector in the projected dimension is a principal component (PC). Each observation is a linear combination of these PCs. The explained variance increases as the rank of the PC increases. PCA is commonly used for data dimensionality reduction. In addition, it has been applied for denoising and signal extraction in image [32], ECG spectra [33], and Raman imaging [34,35] processing. PCA denoising captures and enhances the variances in the spectra that are closely correlated to biomolecules’ changes among observations. In contrast, the traditional denoising algorithms that are commonly used in Raman spectra processing, such as kernel smoothing and Savitzky–Golay differentiation, tend to erase the key features in the spectra. The primary PCs with the biomolecular information are selected and the rest of PCs that mainly contain the random noise are discarded. High-quality spectra and Raman images are reconstructed based on the primary PCs.

The univariate analysis of the signature biomolecules’ Raman band intensities was further conducted. In order to eliminate the impact of the Raman signal intensity difference caused by the background signals from the PBS buffer, the peak normalization based on the PBS buffer signature peak from 910 cm${}^{-1}$ to 920 cm${}^{-1}$ was applied to the dataset. The potential signature biomolecules’ Raman bands of mammalian cells were identified, and the semi-quantification with univariate analysis based on the bands’ intensities was conducted to statistically analyze the biomolecule differences between cell lines.

### 2.4. Machine Learning Models and Predictions

The machine learning classification was further conducted to differentiate the spectra collected from B2B12 and C2C12 cells. The collected Raman dataset was a high-dimensional dataset that consisted of 7122 observations, each having 1022 correlated variables. This dataset showed a low signal-to-noise ratio. Moreover, such a high-dimensional dataset with correlated input features would occupy a large amount of computational resources and lead to the poor performance of the machine learning models. Thereby, the dimensionality reduction including PCA and t-distributed stochastic neighbor embedding (t-SNE) was applied to concentrate the dataset into lower dimensions. The primary PCs were identified based on the PC loadings and the PCA scree plot. Unlike PCA, which projects the original dataset into independent spatial spaces, t-SNE concentrates the high-dimensional dataset into a 2D space through minimizing the Kullback–Leibler divergence between the two probability distributions in the resulting 2D space [36]. The PCA and t-SNE were implemented with R packages “stats” and “Rtsne”. The dimensionality reduction was conducted based on 1650 spectra from B16F10 cells, 880 spectra from C2C12 cells, and 4592 spectra from the PBS buffer background. The resulting data points from B16F1O and C2C12 were employed for machine learning model training.

The dataset of the first 15 primary PCs from PCA and the 2D dataset from t-SNE were used as the input features for machine learning model training. The performances of models’ prediction accuracy based on these two dimensionality reduction algorithms were compared. Each dataset with 2539 observations from B16F10 and C2C12 cells was randomly split into a training set and testing set with a ratio of 3:1, with 1898 observations in the training set and 632 observations in the testing set. The machine learning models were built based on the training set, and model performance was evaluated by the testing set. The model building was achieved with 10-fold cross-validation and 5 repetitions for each model. The classifiers employed for model training included radial basis function support vector machine (SVMRBF), linear support vector machine (SVMLin), random forest (RF), linear discriminant analysis (LDA), quadratic discriminant analysis (QDA), partial least-squares (PLS), k-nearest neighbors (KNN), neural network (NNET), multilayer perceptron (MLP), and naive Bayes (NB). The SVM separates data using hyperplanes in high-dimensional space with different kernel methods. Here, both linear (SVMLin) and radial basis function kernels (SVMRBFs) were used with SVM [37]. RF is an ensemble method to construct multiple decision trees for classification [38]. LDA is a method that classifies or characterizes classes of objectives through linear combinations of features [39]. DQA is more flexible than LDA and does not require a linear decision boundary like LDA [39]. PLS projects the observable variables and predicted variables to a new space to find the linear regression model [40]. KNN is a non-parametric method that calculates the Euclidean distance and feature similarity between stored training data and new input [39]. The NNET performs the classification through an artificial neural network composed of artificial neurons or nodes based on the weight between nodes [39]. MLP is a class of feedforward artificial neural networks that employs a nonlinear activation function [41]. NB is based on Bayes’ theorem with the independent assumptions between features. The model performances were further evaluated, and the sensitivity, specificity, and accuracy were calculated.

## 3. Results and discussion

### 3.1. Identification of Potential Raman Signature Bands

The biomolecule content of cells was identified and quantified from the spectrum through univariate analysis with Raman signature bands. Raman imaging can be built to quantitatively illustrate the biomolecules’ distribution in mammalian cells. The detailed Raman bands’ assignments of typical mammalian cells are given in Table 1.

**Table 1:** Conventional Raman bands’ assignments of mammalian cells.

Wavenumber (cm${}^{-1}$)	Bands’ Assignment
719	Phospholipid (choline) [42]
749	Nucleic acids, Trp
825	Lactic acid
858	Glycans, N-acetyloglucosamine, O-S-O (GAG), glycogen
895	Glycans
917	C-C stretching of proline, glucose, lactic acid [43]
925	Glycans, glycogen, N-acetyloglucosamine
1003	Phenylalanine [44], symmetric ring breathing of protein [45]
1064	Lipids/collagen [46,47] C-C str
1091	Phospholipids [46], O-P-O symmetric stretching,
	P=O symmetric vibration from nucleic acids/cell membrane
	phospholipids
1126	Cytochrome C
1304	Lipids, phospholipids [46] C-H_2_ twist, collagen, protein amide III, DNA [43]
1340	Amide III; CH vibrations (CH_2_ and CH_3_ wagging) of proteins;
	C-C stretching of aromatic ring (proteins);
	Melanin (C-C stretching of aromatic ring and C-H bending—broadband);
	Nucleic acids (guanine); actin [48]
1451	Proteins [46] C-H wag, CH_2_, or CH_3_ def. phospholipids, CH_2_ scissoring [49]
1580	Adenine, guanine (DNA and RNA base) [50]
1651	(C=C) stretching, unsaturated fatty acids, triglycerides
1656	(C=C) stretching [51], Amide I α-helix (amino acids)

### 3.2. Purification and Reconstruction of the Raman Dataset

**Figure 2:**
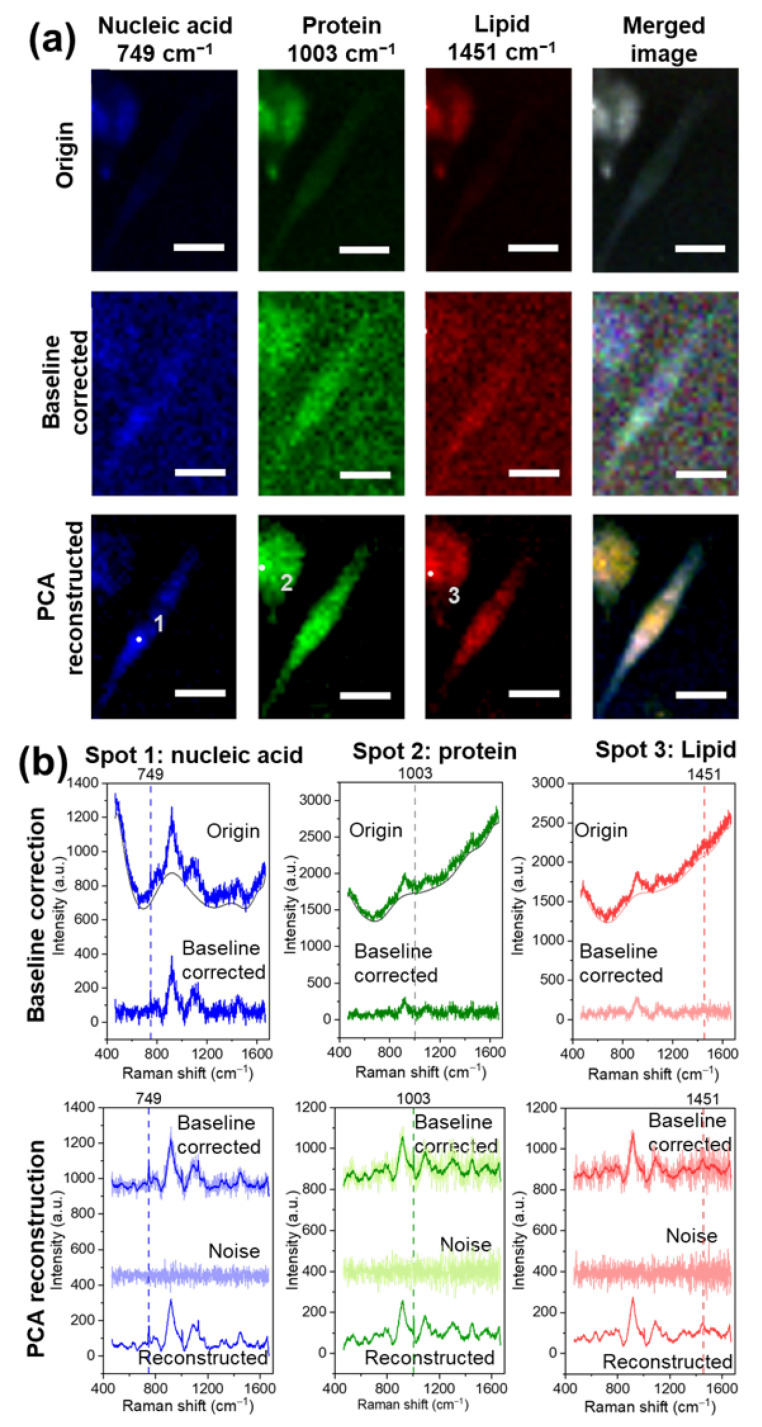
(**a**) Univariate Raman images of B16F10 cells based on Raman signature bands of nucleic acids (749 cm${}^{-1}$), proteins (1003 cm${}^{-1}$), and lipids (1451 cm${}^{-1}$) of the original dataset, after baseline correction, and after PCA denoising. (**b**) Data purification process of typical Raman spectra at spots with high nucleic acid (Spot 1), protein (Spot 2), and lipid (Spot 3) content. The scale bars in the figure above represent 20 μm.

The Raman dataset purification and Raman image reconstruction process is demonstrated with a typical Raman image collected from the cancer cells. The area of interest on the cell-seeded glass was scanned with Raman spectroscopy. The obtained hyperspectral Raman imaging dataset of B16F10 cells consisted of 31 × 43 pixels with a 2 μm scanning step, each pixel containing a Raman spectrum of the examined pixel that covered the area of 4 μm${}^{2}$. The univariate imaging (Figure 2a) of the dataset was first performed to demonstrate the performance of the data purification and reconstruction process. The univariate images were built based on distinct Raman bands’ intensities of nucleic acids (749 cm${}^{-1}$), proteins (1003 cm${}^{-1}$), and lipids (1448 cm${}^{-1}$). The brightness of each pixel in the Raman images represent the signature peak intensity of the biomolecules of interest at the spot where the spectrum was collected. The brighter the pixel is in the picture, the higher the signature peak intensity of the biomolecule is, which reflects the higher content of such biomolecules in the designated area. The typical spots with a high content of signature biomolecules nucleic acids (Spot 1), proteins (Spot 2), and lipids (Spot 3) are marked on Figure 2a, and the spectra purification process at marked spots is shown in Figure 2b. As shown in Figure 2b, the origin spectra were inflated and distorted by the background fluorescence, and the random noise masked the signature bands of typical biomolecules of mammalian cells, nucleic acids (749 cm${}^{-1}$), proteins (1003 cm${}^{-1}$), and lipids (1448 cm${}^{-1}$). The corresponding Raman images of nucleic acids, proteins, and lipids (Figure 2a) were largely affected by the fluorescence. The area with stronger background fluorescence (such as Spots 2 and 3) was brighter, while the area with lower background fluorescence (such as Spot 1) was darker. As a consequence, the cell at the lower right in the original Raman image is almost invisible. The background noise originated from multiple sources, including Stokes shift fluorescence and the sample substrate. The fluorescence shift can vary from sample to sample and even spot to spot.

In order to eliminate the effect of fluorescence, the polynomial baseline correction (degree of polynomial: 9, tolerance of difference between iterations: 0.001, the maximum number of iterations: 100) was performed. Polynomial fitting was selected due to its straightforward and convenient nature for baseline correction. It requires minimal information input and yields optimal fitting results. A unique polynomial curve was customized to fit below each spectrum to remove the background, as shown in Figure 2b. The baseline was subsequently subtracted from each spectrum. The removal of the high-intensity background led to a low signal-to-noise ratio because the Raman signal was weakened after the baseline substation. Thereby, the corresponding Raman imaging was strongly affected by the random noise after the baseline correction. Moreover, the resulting Raman images showed blurry boundaries between cells and the background, as shown in Figure 2a.

To separate the Raman signals that reflected the biomolecules’ content changes from the random noise in the spectra dataset, the PCA reconstruction process was applied to the pre-processed dataset. The PCs that caused the primary variances were identified to reconstruct the Raman spectra. The PC loadings of the first 20 PCs are shown in Figure S2. The first 17 PCs contained the information associated with the Raman bands of the mammalian cells’ signature biomolecules listed in Table 1. The rest of the PCs consisted of random noise. Moreover, the PC scree plot of the first 20 PCs presented the variance explained by the first few PCs, as shown in Figure S3. The elbow point of the scree plot was at the 17th PC, and the first 17 PCs explained the majority of the variance. Thereby, the first 17 PCs were selected to reconstruct the Raman dataset. As shown in Figure 2, the reconstructed spectra showed clear signature bands of nucleic acids (749 cm${}^{-1}$), proteins (1003 cm${}^{-1}$), and lipids (1451 cm${}^{-1}$), respectively. The spectra constructed based on the PCs that mainly contained the random noise were labeled as noise (Figure 2b). In contrast, the constructed noise spectra showed no sign of Raman bands. As shown in Figure 2, the dominant Raman band of the blue spectrum collected at Spot 1 was the nucleic acid signature band at 749 cm${}^{-1}$, while the protein band at 1003 cm${}^{-1}$ was weak. The green spectrum at Spot 2 showed the dominant peak of proteins at 1003 cm${}^{-1}$, while the nucleic acid band at 749 cm${}^{-1}$ was weak and difficult to identify. The red spectra collected from Spot 3 showed the clear lipid signature band at 1451 cm${}^{-1}$; the same lipid band can also be identified at spectra collected from Spot 1 (blue) and Spot 2 (green). The corresponding univariate Raman images after PCA denoising are shown in Figure 2a. The random noise in the background was filtered out based on the Raman bands’ threshold intensities. The reconstructed Raman images showed clear boundaries between Raman cells and the background, and the distribution of the biomolecules can be identified. The tumor cells can be classified into three predominant morphologies: elongated morphology, round morphology, and a mixture of both. The different mythologies allow the cancer cells to invade into various microenvironments [52]. The merged Raman image of nucleic acids, proteins, and lipids showed the morphology of the cells and the chemical images of these molecules. As shown in Figure 2a, the cell at the lower right is an elongated cancer cell, while the cell at the upper left is a cell between a round and elongated morphology. The typical optical images, baseline-corrected Raman images, and PCA-reconstructed Raman images based on protein signature peak intensity at 1003 cm${}^{-1}$ of both cancer cells (B16F10) and non-cancer cells (C2C12) are shown in Figure S4.

### 3.3. Comparison of PCA with Traditional Denoising Algorithms

The PCA denoising result is compared with two commonly used denoising algorithms for the Raman dataset, kernel smoothing [53] and Savitzky–Golay differentiation [54].

**Figure 3:**
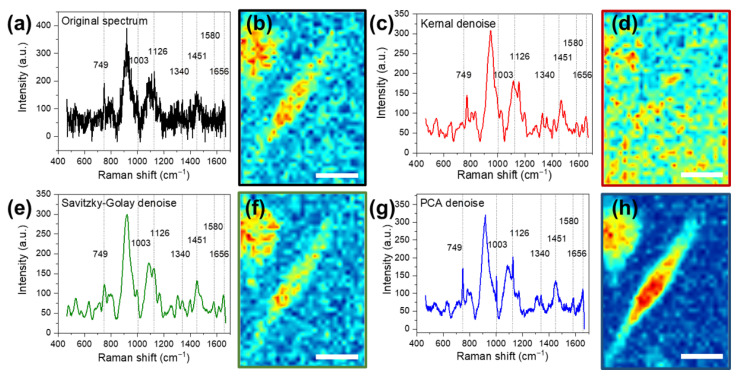
Typical Raman spectra and corresponding Raman images based on the protein Raman signature band at 1003 cm${}^{-1}$ (**a**,**b**) without processing, (**c**,**d**) with kernel denoising, (**e**,**f**) with Savitzky–Golay denoising, and (**g**,**h**) with PCA denoising. The scale bars in the figure above represent 20 μm.

As shown in Figure 3, a typical Raman spectrum (Figure 3a) was selected from the dataset; the same spectrum after kernel smoothing (Figure 3c), Savitzky–Golay differentiation (Figure 3e), and PCA denoising (Figure 3g) are shown. The signature Raman bands of the biomolecules are marked with dashed lines, and bands’ positions are labeled. The corresponding Raman images of the dataset were constructed based on the protein band intensity at 1003 cm${}^{-1}$, as shown in Figure 3b,d,f,h, respectively. The pseudo heat map images of the Raman band illustrate the band intensities at the pixels; the area with red color shows higher band intensity, while the area with blue color represents the lower band intensity. The Raman bands of the original spectrum were masked by the noise; both the band position and band intensity cannot be accurately identified (Figure 3a). The resulting Raman image was noisy with blurry boundaries between cells and PBS buffer background (Figure 3b). The kernel smoothing denoising algorithm (kernel: normal, bandwidth: 10) was conducted to smooth the spectrum. The processed Raman spectrum showed a higher SNR; however, the resulting Raman bands were largely shifted from the known signature Raman bands of the biomolecules (Figure 3c). Consequently, the cells can hardly be identified in the resulting Raman images (Figure 3d). The Savitzky–Golay differentiation-processed (filter length: 51, filter order: 4, derivative order: 0) spectrum showed a higher SNR and accurate signature band positions (Figure 3e). However, it tended to smooth out the sharp signature bands. The resulting corresponding Raman image shows no sign of quality improvement (Figure 3f). The PCA denoising-processed spectrum showed a high SNR and accurate band positions (Figure 3g). Moreover, the sharp signature bands of nucleic acids (749 cm${}^{-1}$) and proteins (1003 cm${}^{-1}$) were both well retained. The corresponding Raman image (Figure 3h) quality was significantly improved. The cell area is highlighted, and the boundaries between cells and background PBS buffer are clearly shown. Thereby, the commonly used smoothing algorithms showed limited ability to process the low-quality dataset. In contrast, PCA denoising can successfully extract and highlight the biomolecules’ information related to the cell type differences. This character is crucial both for univariate analysis and subsequent machine learning differentiation.

### 3.4. Univariate Analysis of Biomolecules’ Content

**Figure 4:**
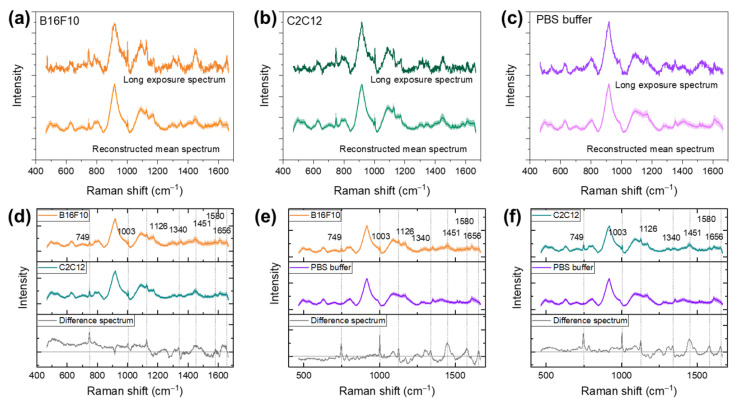
MeanRaman spectra of the PCA-reconstructed dataset and spectrum collected with long acquisition time for (**a**) B16F10 cells, (**b**) C2C12 cells, and (**c**) PBS buffer background. The different spectra between (**d**) B16F10 and PBS buffer, (**e**) C2C12 and PBS buffer, and (**f**) B16F10 and C2C12.

The mean spectra of B16F10, C2C12, and background PBS buffer were calculated to further study the biomolecules’ content differences for these three groups. The Raman spectra collected from B16F10, C2C12, and background PBS buffer were manually labeled based on the optical images collected during Raman imaging. The baseline-corrected dataset was further normalized based on the integral of the PBS buffer signature peak at 910 cm${}^{-1}$ to 920 cm${}^{-1}$ to eliminate the effect of the PBS buffer background. The spectra were reconstructed with the PCA denoising methods mentioned previously. The mean spectra were compared with the high-quality spectra collected with a long acquisition time. A 20 s acquisition time was set to collect high-quality Raman spectra, and the excitation laser power was set to be 50 mW to prevent the heat damaging of cells caused by overly high laser power. The reconstructed mean spectra with variance labels in a lighter shade of color and spectra with a long exposure time were rescaled to the same magnitude and shown in Figure 4a–c. The high-quality Raman spectra showed visible random noise even with a 20-times longer exposure time compared with the reconstruction spectra of the fast Raman imaging spectrum acquisition setting. The reconstructed mean spectra of all cells and PBS buffer showed good recovery and a high SNR. Both B16F10 and C2C12 mean spectra and high-quality Raman spectra showed distinct Raman bands designated to mammalian cells’ signature biomolecules listed in Table 1. On the other hand, the mean PBS buffer spectrum showed no sign of biomolecules’ signature bands.

The mean difference spectra of B16F10, C2C12, and PBS buffer are shown in Figure 4d–f. The Raman bands that were designated to biomolecules are marked with the dashed lines, and the band positions are labeled. The difference spectra of B16F10 and PBS buffer (Figure 4d) and C2C12 and PBS buffer (Figure 4e) showed a significant difference of the conventional Raman bands of the biomolecules. This specifically illustrates the content variance of phospholipids (1126, 1451 cm${}^{-1}$), nucleic acids (749, 1340 cm${}^{-1}$), phenylalanine of proteins (1003 cm${}^{-1}$), Cytochrome C (1126 cm${}^{-1}$) and collagen, and protein Amide III (1342 cm${}^{-1}$) between the B16F10 and C2C12 cell lines and PBS buffer. Moreover, there were no significant PBS buffer signature bands at 634, 808, and 916 cm${}^{-1}$ shown in the difference spectra. This proves that the PBS buffer background Raman bands were eliminated in the difference spectra. The difference spectra of B16F10 and C2C12 (Figure 4f) showed the Raman bands’ intensity difference of signature biomolecules between these cell lines.

**Figure 5:**
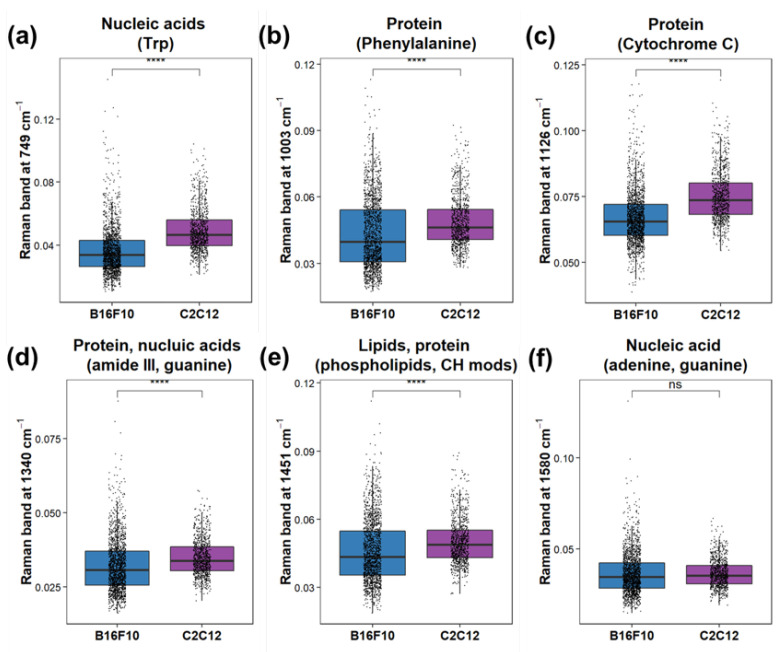
ANOVA test of band intensities of mammalian cells for B16F10 and C2C12 at (**a**) 749 cm${}^{-1}$ resulting from nucleic acid (Trp), (**b**) 1003 cm${}^{-1}$ resulting from protein (phenylalanine), (**c**) 1126 cm${}^{-1}$ resulting from Cytochrome C, (**d**) 1340 cm${}^{-1}$ resulting from Amide III, (**e**) 1451 cm${}^{-1}$ resulting from lipid phospholipid, and (**f**) 1580 cm${}^{-1}$ resulting from nucleic acids (adenine, guanine). **** represents *p* ≤ 0.0001, ns represents not significant.

In order to confirm the biomolecules’ differences between B16F10 and C2C12 cells, a semi-quantification of biomolecules’ Raman bands is conducted as shown in Figure 5. The nucleic acid Raman band at 749 cm${}^{-1}$ (Figure 5a) was significantly lower (*p* ≤ 0.0001) for B16F10 compared to C2C12, while the nucleic acid band resulting from adenine and guanine at 1580 cm${}^{-1}$ (Figure 5f) showed no significant difference. The protein Raman bands at 1003 cm${}^{-1}$ resulted from phenylalanine (Figure 5b); 1126 cm${}^{-1}$ resulted from Cytochrome C (Figure 5c); 1340 cm${}^{-1}$ (Figure 5d) resulted from Amide III; these were significantly lower (*p* ≤ 0.0001) for B16F10 compared to C2C12. The Raman band of lipid phospholipids at 1451 cm${}^{-1}$ also showed significantly lower (*p* ≤ 0.0001) intensity for B16F10 compared to C2C12.

### 3.5. Machine Learning Classification

**Figure 6:**
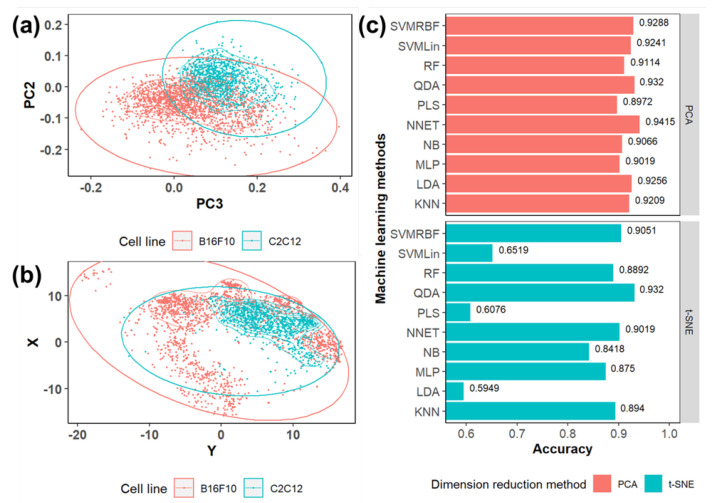
Two-dimensional visualization of the (**a**) PCA plot of PC2 vs. PC3 and (**b**) 2D t−SNE plot for data points from B16F10 and C2C12. (**c**) The prediction accuracy of different machine learning models to differentiate data collected from B16F10 and C2C12 based on PCA and t−SNE dimensionality reduction algorithms, respectively.

The previous univariate analysis was focused on the individual Raman bands, but it is not sufficient to study the correlations between biomolecules and extract the spectral information that is crucial for biological systems. Moreover, it is time consuming and difficult to manually differentiate B16F10 and C2C12 cells by the univariate analysis, especially with a large dataset. Thereby, the machine learning model building and cell type differentiation were further conducted. The multivariate dimension-reducing algorithms such as PCA and t-SNE were applied to the previous baseline-corrected, normalized, and labeled dataset. The dimensionality reduction algorithm can significantly reduce the computation time and resource consumption by reducing the dimension of the original dataset through highlighting the different features within the dataset. The PCA dimensionality reduction was conducted, and the primary PCswere picked out with the same process mentioned previously based on the PC loading and the scree plot of PCA. The first 15 PCs of PCA and 2D t-SNE were used for machine learning model building. Additionally, the PCA plot and t-SNE plot were the tools to visualize the data distribution in lower dimensions. The PC2 against PC3 plot (Figure 6a) and t-SNE plot (Figure 6b) were used to visualize the distribution of cell spectra in the 2D space. The PCs were ranked based on the variance that was explained by the PC: PC1 explains most of the variance. However, although PC1 can help separate the sample groups in a higher dimensional space, the PC1 vs. PC2 plot could not successfully separate the sample groups in the 2D space. Thereby, the PC2 vs. PC3 plot, which showed better sample group separation in the 2D space, was selected to visualize the sample groups’ distribution in the 2D space. The points representing the data collected from B16F10 cells are in red and those from C2C12 in green; the data clusters are further highlighted with the red and green circles. The PCA plot shows the tight cluster of points from B16F10 and C2C12 cell lines with some overlap, while the data points from B16F10 are more scattered in the t-SNE plot.

Different machine learning algorithms were further used to build the models, including SVMRBF, SVMLin, RF, LDA, QDA, PLS, KNN, NNET, MLP, and NB classifications. The 10 different models were trained individually based on the PCA-reduced dataset and t-SNE-reduced dataset, respectively. The machine learning prediction results based on different dimensionality reduction algorithms are shown in Figure 6c. With the PCA dimensionality reduction, the prediction accuracy rates showed good consistency between different algorithms, the best prediction accuracy of 94.15% being achieved by the NNET classification, while the prediction accuracy of all algorithms was higher than 90%, except for PLS. On the other hand, the machine learning prediction accuracy based on the t-SNE algorithm showed significant variance between different algorithms, the highest accuracy rate being 93.2% with the QDA; however, SVMLin, PLS, and LDA yielded a accuracy rate of only around 60%. The machine learning models based on PCA dimensionality reduction yielded overall higher accuracy and better consistency compared with the t-SNE algorithm. The accuracy and the robustness of the models can be further improved by increasing the training data size and further tuning of the models.

Finally, the classification accuracy of machine-learning-assisted Raman spectroscopy for cancer detection was compared with similar techniques that have been recently published in the research literature (Table 2). The data acquisition time of our work was significantly lower than other works with an excellent accuracy rate. While compared with other research for skin cancer detection [55,56,57], our work showed the second-best accuracy and well-balanced sensitivity and specificity. Compared with the research with single cell differentiation based on cell cultures, our work showed excellent accuracy (94.15% accuracy) compared with the work with a short acquisition time of 2 s (89.6% accuracy) [58] and comparable accuracy with the work with an extended acquisition time of 60 s (92–96% accuracy) [59]. Thereby, our method is time-efficient and robust compared with other machine-learning-assisted Raman spectroscopy for cancer detection.

## 4. Conclusions

In this study, a machine-learning-assisted fast Raman imaging process was successfully developed for living cancer and healthy murine cells’ imaging and differentiation. The fast imaging method requires minimal sample preparation and is capable of collecting Raman signals from living samples in a non-invasive manner. The denoising and dimension reduction algorithm based on PCA was applied to recover the signal variation information from the low-quality Raman signal collected with a short acquisition time. The resulting recovered high-quality Raman spectra showed a significantly higher SNR. The Raman images constructed based on the signature biomolecule bands showed lower noise and successfully reflected the morphology and the biomolecule contents’ distribution of the cells. The traditional Raman spectra denoising algorithms such as kernel denoising and Savitzky–Golay denoising tended to distort the peak position and/or the peak intensity. On the contrary, PCA denoising accurately retained the peak position and intensity to significantly improve the quality of both the spectra and corresponding Raman images. The Raman dataset was further utilized for the semi-quantification of signature biomolecules’ Raman bands’ intensities and the machine learning models’ training. Another commonly used dimensionality reduction algorithm was employed in comparison with PCA. The machine learning classifiers including the SVMRBF, SVMLin, RF, LDA, QDA, PLS, KNN, NNET, MLP, and NB classifiers were used for model training. The prediction accuracies of machine learning models based on PCA or t-SNE dimensionality reduction were compared. The best prediction accuracy of 94.15% was achieved by the neural network (NNET) classification based on PCA dimensionality reduction. Moreover, the prediction accuracy of machine learning models based PCA dimensionality reduction showed higher consistency and accuracy compared with the t-SNE algorithm. The fast Raman imaging and data processing techniques developed here might be used for in situ or in vivo live cell monitoring or cancer diagnosis.

## Figures and Tables

**Table 2:** Performance comparison of cancer detection with machine learning and Raman spectroscopy.

Sample	Targeted Cancer	Acquisition Time	Accuracy	Sensitivity	Specificity	Ref
		(s)	(%)	(%)	(%)	
skin tissue	skin cancer	20	in vivo 93.8	94.1	93.8	[55]
	skin cancer		ex vivo 100	100	100	
skin tissue	skin cancer	30	NA	45	100	[56]
tissue block	skin cancer	NA	NA	100	84	[57]
cell culture	skin cancer	1	94.15	94.17	94.09	this work
cell culture	breast cancer	200	100	100	100	[60]
cell culture	lung cancer	2	89.6	NA	NA	[58]
cancer tissue	kidney cancer	5	81.4	NA	NA	
cell culture	cervical cancer	60	NA	>95	>92	[59]

NA represent the information is not available.

## Data Availability

The data that supported the findings of this study are openly available in Mendeley Data at http://doi.org/10.17632/yzfy4xhfy7.1, accessed on 15 March 2022.

## Supplementary Materials

The following are available online at https://www.mdpi.com/article/10.3390/bios12040250/s1, Figure S1: Optical images of cancer cells and healthy cells. Figure S2: Raman spectra of the cells’: (1) baseline corrected dataset; and (2) PCA reconstructed dataset. Figure S3: PCA scree plot. Figure S4: Optical images of (a) cancer cells (B16F10) and (d) healthy cells (C2C12), the corresponding Raman images (based on protein signature peak intensity at 1003 cm${}^{-1}$) of (b) cancer cells (B16F10) and (e) healthy cells (C2C12) after baseline correction, and the corresponding PCA-reconstructed Raman images (based on protein signature peak intensity at 1003 cm${}^{-1}$) of (c) cancer cells (B16F10) and (f) healthy cells (C2C12). Figure S5: Raman spectra of C2C12 cells with a 5, 10, 20, and 30 s acquisition time. Table S1: Classification of B16F10 and C2C12 cells by machine learning models based on different dimension reduction algorithms.
