# Supplementary material for: Label-Free Differentiation of Cancer and Non-Cancer Cells Based on Machine-Learning-Algorithm-Assisted Fast Raman Imaging
Source: Biosensors (Basel). 2022 Apr 15;12(4):250. doi: 10.3390/bios12040250 (PMC9031282; doi:10.3390/bios12040250)
Supplement: Supplementary file 1 [file biosensors-12-00250-s001.zip › biosensors-1662021-supplementary.pdf]

Supplementary Materials: Label-Free Differentiation of Cancer and Non-Cancer Cells Based on Machine-Learning-Algorithm-Assisted Fast Raman Imaging

Qing He [1,*,†,‡], Wen Yang [2,†], Weiquan Luo [3,§], Stefan Wilhelm [2] and Binbin Weng [1,*]

Contents

0.1. Optical images of cells . . . 1
0.2. PCA reconstruction . . . 1
0.3. Primary PCs identification . . . 2
0.4. PCA reconstructed Raman images of cancer and healthy cells . . . 3
0.5. Optimal acquisition time for single spectrum collection . . . 3
0.6. Machine learning prediction with PCA or t-SNE . . . 4

0.1. Optical images of cells

The optical images of cancer cells (B16F10) and non-cancer cells (C2C12) with 4X, 10X and 20X magnifications are shown in Figure S1.

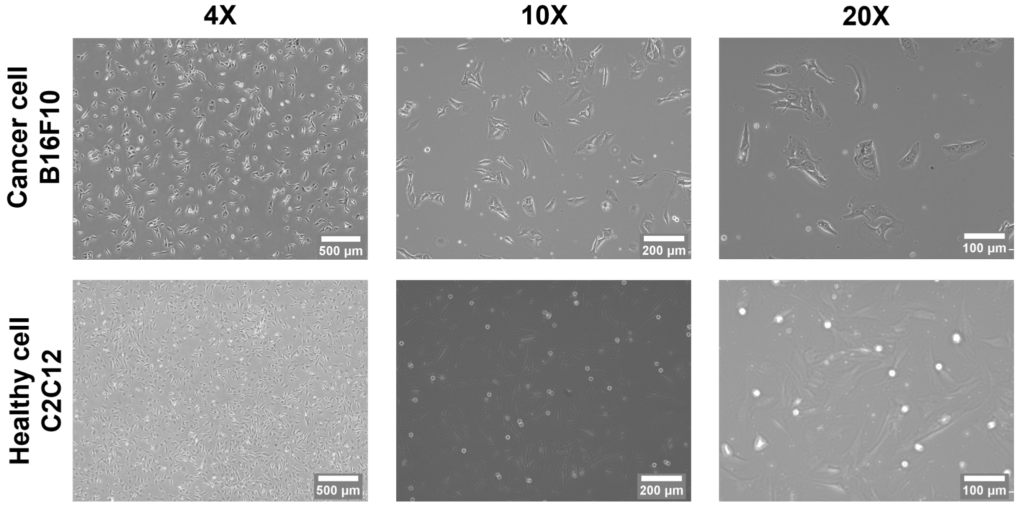

Figure S1. Optical images of cells cancer and non-cancer cells.

0.2. PCA reconstruction

The original dataset is pre-processed to first remove the comic ray, and correct the effect background fluorescent. The pre-processed dataset **X** is a matrix that consists of n spectra collected from the scanned pixels and each spectrum with p intensity values at corresponding wavenumber of the spectrum. The PCA process decomposes the **X** into follow:

$$X = hu^T + DV$$

Where **h** is an $n \times 1$ column vectors of 1, u^T is the mean spectrum of all observations. **V** denotes the matrix of eigenvectors, where each column is a PC loading, **D** is the matrix of corresponding eigenvalues. The Raman dataset is then reconstructed by the first few PCs that explain the most variance. In specific, the subset of **D** and **V** that contains the first ith PCs and corresponding eignvalues, D' V', are used to reconstruct the new dataset X' as follow:

$$X' = hu^T + D'V'$$

The rest of the PCs that mainly contain the random noises are discarded.

0.3. Primary PCs identification

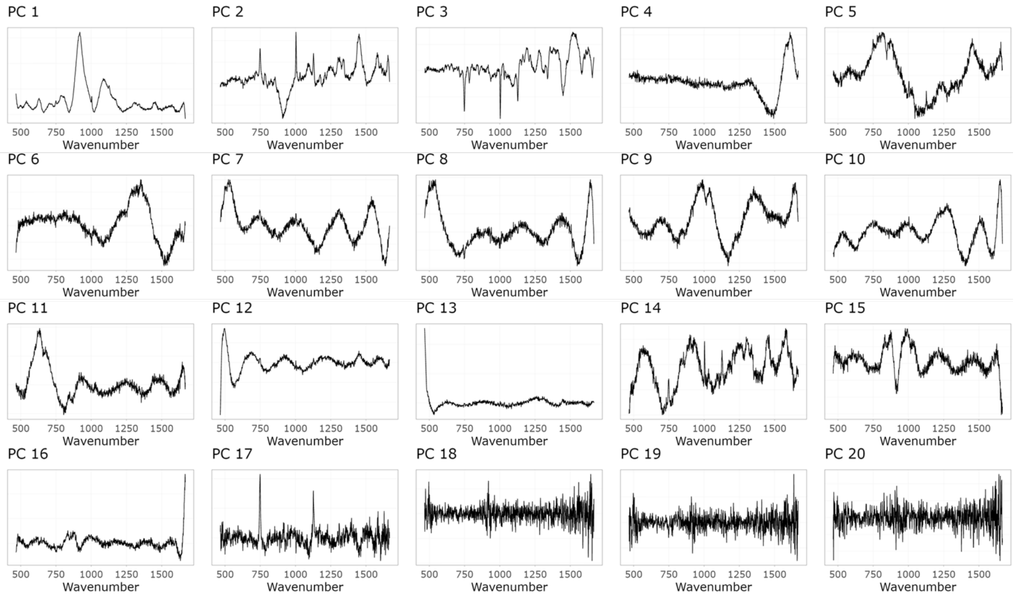

Figure S2. Raman spectra of the cells 1) Baseline corrected data set; and 2) PCA reconstructed data set.

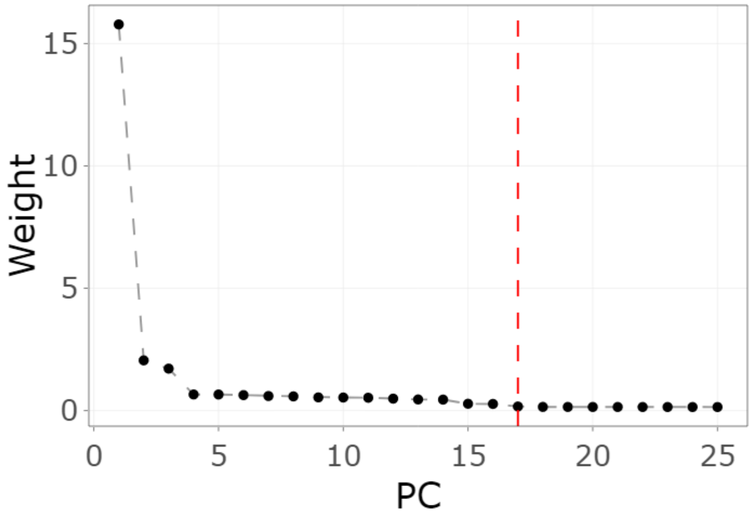

Figure S3. PCA scree plot.

The primary PCs are manually identified based on the PC loadings and the PCA scree plot. The PC loadings of the first 20 PCs are shown in Figure S1, the first 17 PCs contains the information associated with the Raman bands of the signature biomolecules of mammalian cells. The rest of the PCs are consists of random noise. The PC scree plot is further considered for primary PCs identification. The PC scree plot shows the variance explained by PCs. As shown in Figure S2, the elbow point of the scree plot is at the 17th PC. The first 17th PCs explain majority of the variance. Thereby, the first 17th PCs reflect the biomolecules content change are selected to reconstruct the Raman dataset.

0.4. PCA reconstructed Raman images of cancer and healthy cells

The optical images of cancer cells (B16F10) and healthy cells (C2C12) (Figure S4 (a), (d)), the corresponding Raman images based on protein signature peak intensity at 1003 cm^{-1} after the baseline correction of the spectra (Figure S4 (b),(e)) and after the PCA reconstructions (Figure S4 (c),(f)). As shown in the figure, after the baseline correction process of the spectra, the background noise is high, thereby, the boundary between cells and the background is blurry in the Raman images. After the PCA reconstruction, the Raman image quality improved significantly. The noise in the background is lower and the boundary between cells and the background and easy to be identified.

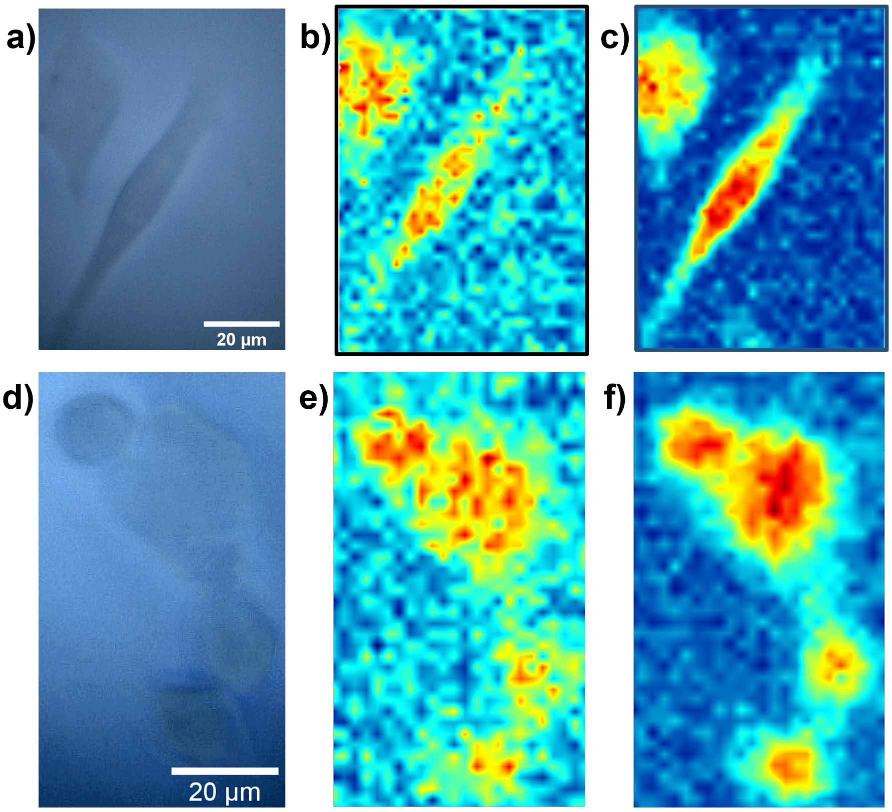

Figure S4. Optical images of a) cancer cells (B16F10) and d) healthy cells (C2C12), the corresponding Raman images (based on protein signature peak intensity at 1003 cm^{-1}) of b) cancer cells (B16F10) and e) healthy cells (C2C12) after baseline correction, and the corresponding PCA reconstructed Raman images (based on protein signature peak intensity at 1003 cm^{-1}) of c) cancer cells (B16F10) and f) healthy cells (C2C12).

0.5. Optimal acquisition time for single spectrum collection

The Optimal acquisition time for single spectrum collection was conducted by collecting the Raman spectra from the same C2C12 sample with different acquisition time of 5, 10, 20 and 30 seconds. The optimal acquisition time was select when the Raman spectra signal to noise ratio no long improve with the acquisition time increases. The spectra collected with different acquisition time are normalized based on maximum peak intensity of the spectra. As shown in Figure S5, with the 5 second acquisition time, the Raman peaks of Trp for nucleic acid of at 749 cm^{-1} and phenylalanine for protein at 1003 cm^{-1} can be identified. However, the signiture peaks of Cytochrom C for protein at 1126 cm^{-1} and some protein and lipids signature peaks at 1451 cm^{-1} and 1580 cm^{-1} can be hardly identified from the spectrum. With the acquisition time increase from 5 second to 20 second, the signal to noise ratio of the spectra gradually increases, while the signal to noise ratio of spectra collected with 20 seconds and 30 seconds don't show significant differences. Thereby, 20 second

acquisition time is selected for single spectra acquisition for the rest of the experiment to efficiently obtain high quality spectra.

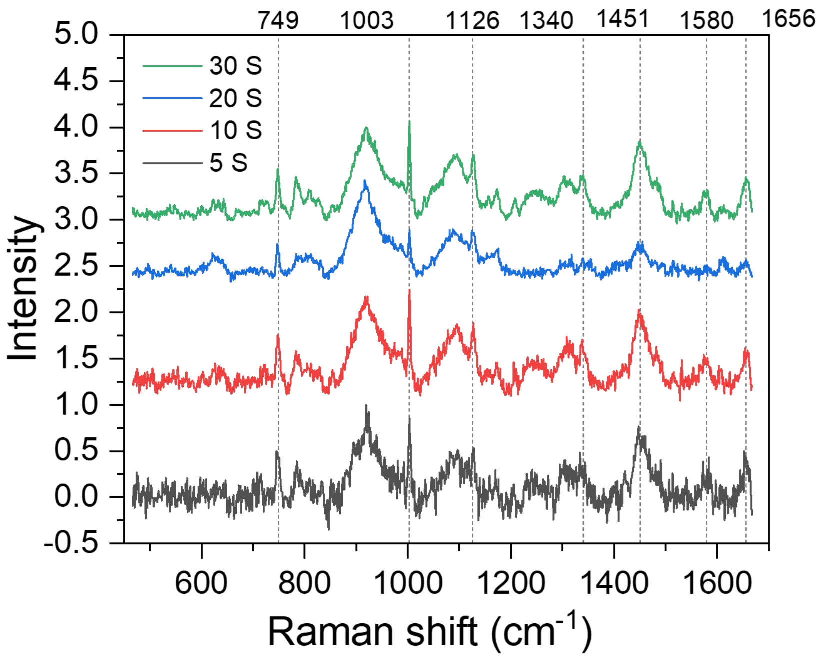

Figure S5. Raman spectra of C2C12 cell with 5, 10, 20, 30 seconds acquisition time

0.6. Machine learning prediction with PCA or t-SNE

The confusion matrix of the machine learning model predictions are further calculated, and the key parameters including accuracy, sensitivity, and specificity are shown in Table S1. The accuracy, sensitivity and specificity are calculated as below:

$$Accuracy = (TP + TN)/TP + TN + FP + FN$$

$$Sensitivity = TP/(TP + FN)$$

$$Specificity = TN/(TN + FP)$$

where TP is true positive number, FP is false positive number, TN is true negative number, and FN is false negative number.

Table S1. Classification of B16F10 and C2C12 cells by machine learning models based on different dimension reduction algorithms

ML algorithm	DR algorithm	Accuracy	Sensitivity	Specificity
KNN	PCA	92.09	91.99	92.27
	t-SNE	89.4	89.56	89.09
LDA	PCA	92.56	92.23	93.18
	t-SNE	59.49	76.7	27.27
MLP	PCA	90.19	97.82	75.91
	t-SNE	87.5	90.05	82.73
NB	PCA	90.66	92.96	86.36
	t-SNE	84.18	86.65	79.55
NNET	PCA	94.15	94.17	94.09
	t-SNE	90.19	89.56	91.36
PLS	PCA	89.72	93.2	83.18
	t-SNE	60.76	79.61	25.45
QDA	PCA	93.2	91.99	95.45
	t-SNE	93.2	91.99	95.45
RF	PCA	91.14	93.93	85.91
	t-SNE	88.92	88.59	89.55
SVMLin	PCA	92.41	92.23	92.73
	t-SNE	65.19	100	0
SVMRBF	PCA	92.88	92.23	94.09
	t-SNE	90.51	89.81	91.82
